# Fungi at a Small Scale: Spatial Zonation of Fungal Assemblages around Single Trees

**DOI:** 10.1371/journal.pone.0078295

**Published:** 2013-10-16

**Authors:** Sara Branco, Thomas D. Bruns, Ian Singleton

**Affiliations:** 1 Department of Plant and Microbial Biology, University of California, Berkeley, California, United States of America; 2 School of Biology and Newcastle Institute for Research on Sustainability, Newcastle University, Newcastle upon Tyne, United Kingdom; University of Alberta, Canada

## Abstract

Biological communities are often structured by environmental factors even at small spatial scales. Fungi are no exception, though the patterns and mechanisms underlying their community structure are usually unknown. Previous work documented zonation in fungi under tree canopies primarily through their fruiting patterns. Here we investigate the existence of zonation patterns in fungal communities around isolated *Pinus muricata* trees of different ages in northern coastal California. Using a combination of ingrowth bags and pyrosequencing to target underground mycelium we found highly diverse soil fungal communities associated with single trees. Both ectomycorrhizal and non-ectomycorrhizal fungi were present in all samples, but the latter were more species rich, dominated the samples by sequence read abundance, and showed partitioning by canopy-defined zones and tree age. Soil chemistry was correlated with fungal zonation, but host root density was not. Our results indicate different guilds of fungi partition space differently and are driven by distinct environmental parameters.

## Introduction

Fungi play crucial roles in ecosystems, namely as decomposition agents, pathogens, and mycorrhizal symbionts, and these different functional fungal groups are most likely influenced by distinctive environmental parameters. Similar to other soil microbes, soil fungal assemblages are typically very diverse, lack clear dominants, and contain many rare species. This trend is recurrent across both saprotrophic [1-3] and mycorrhizal fungi [4-6]. In forested systems ectomycorrhizal (EM) fungal assemblages are known to be structured by disturbance and successional processes [7-10], dispersal [11,12], differences in spore behavior [13-15], competition [16], soil horizon [17,18], soil chemistry [19,20] and plant host [21,22]. While fungal saprotrophic communities have received less recent attention, they seem to be structured by litter succession and soil properties [23], dispersal [24], soil horizon [25], nutrient availability [26], litter type [27], and litter quality [28]. However, this is most likely an incomplete list of factors and it is not clear which are the primary drivers or if they affect spatial distributions for fungi at a local scale.

Here we investigated the spatial zonation structure of soil fungal communities at the scale of single trees. Specifically, we looked for differences in fungal assemblage composition close to the edge of the canopy compared to nearer the stem of the tree. Such zonation has been previously seen either at the community or single species level around forest patches, trees and shrubs [23,29-31]. These patterns were usually based on differences in fruiting pattern or mycorrhizal root tips and have been suggested to be determined by a combination of fungal extrinsic and intrinsic parameters such as soil chemistry [23], host root density [31], and trade-offs in dispersal and competitive abilities across fungi [9,32]. We tested for the existence of soil fungal community zonation around single trees and for differences associated with tree age. Our observations were based on mycelium as assayed with ingrowth bags and high-throughput sequencing. Our hypotheses were: 1) EM and non-EM fungal communities show different spatial patterns across single trees and tree age; 2) EM fungal assemblage structure would be correlated with root density; 3) Conversely, non-EM fungal diversity is dependent on soil properties alone and not expected to vary with canopy zonation or tree host age. 

We expected EM fungi to exhibit clear spatial zonation around single trees since this had been previously observed based on fruit bodies and EM roots [29-31]. Root density gradients correlated with distance from the stem have been recently hypothesized to be one of the driving factors for EM fungi [31], so we expected a correlation between at least EM fungal assemblage structure and root density. Similarly, these and related studies [33] showed differences in EM fungal communities based on tree age, with pioneer species dominating young trees. Saprotrophic fungal communities have been less studied, however at least one species of Mycena was found to have similar pattern of zonation under tree canopies [23], but this pattern was attributed to soil chemistry rather than root density.

Our investigation of zonation differed in two primary ways from past studies: 1) it was based on mycelium rather than fruiting or EM roots, and 2) the fungi present were determined by high-throughput sequence analysis. We sampled fungal mycelium using ingrowth bags, a technique that uses mesh bags filled with acid-washed sand and targets actively growing fungi [6,34]. We sampled two canopy-defined zones under single canopies of young and old trees and assessed the fungal species present by pyrosequencing. In addition, we tested for the effect of sequencing depth in recovering spatial patterns in fungal communities by comparing reads generated from libraries from individual and pooled bags. 

## Materials and Methods

### Study site

This study was carried out at Point Reyes National Seashore (PRNS) situated in west Marin County, California (38°04’ N by 122°50’ W), under a permit issued by the National Park Service (study #67). PRNS has a Mediterranean climate characterized by mild and wet winters and cool and dry summers. The mean average temperature is 11°C, September average is 13.5°C and January average is 10°C. Precipitation averages around 43 cm and is primarily restricted to the winter months. More details on the climate, geography and vegetation of this Mediterranean area can be found in [11]. This part of PRNS is occupied by monodominant stands of *Pinus muricata* D. Don (Bishop pine), the only EM host in the study plots, occurring both as forest patches and as isolated trees across the landscape. The 1995 Mount Vision Fire resulted in a large area of trees that were 16 years old at the time of this study, with an adjacent area of older trees. This setting is ideal for testing the fungal community patterns around single trees and the effect of tree age on fungal communities, as it provides isolated trees of different ages, established before and after the Mount Vision Fire, but in the same soil type and climate.

### Experimental design

We sampled a total of 20 individual trees (‘island’ trees), of which 10 were young (generally 16 years old) and 10 old (trees estimated to be 50 to 80 years old). We selected individual trees whose canopies were not adjacent to other trees. Figure S1 shows a map with all tree locations.

Fungal communities were sampled using sand-filled ingrowth bags that allow for mycelium colonization and were expected to be biased toward sampling of EM fungi [6,34]. We assembled 5x5 cm ingrowth bags using anti-static polyester fabric obtained from a local fabric store. The fabric had an approximate pore size of 50 μm that allowed mycelium to grow through but exclude plant roots. Each bag was filled with 5 ml of acid-washed autoclaved sand (5% v/v HCL overnight; sand was autoclaved three times on three consecutive days) and sealed with a heat sealer. A nylon string was attached to each bag to facilitate retrieval. 

We defined two concentric circles around each tree trunk, one at the edge of the tree canopy or drip line (outer circle) and another at one third of the distance to the canopy (inner circle), and buried 4 ingrowth bags at opposing directions in each circle (N, S, E, and W directions) at 5 to 10 cm depth. The rationale underlying the choice of inner and outer circles was that root density, water availability, and soil chemistry would differ sufficiently to capture potential fungal differences. We buried a total of 160 bags in November of 2011 and collected them 2 months later (January 2012). Bags were stored at 4° and DNA extracted within 7 days of collection. Two bags that were not buried in the field were used as contamination controls for DNA extraction, PCR, and sequencing.

### Molecular protocols for fungal identification

Fungal DNA from the ingrowth bags was extracted, amplified and pyrosequenced. Sand from each bag was emptied into a 50 ml screw cap sterile disposable centrifuge tube, 10 ml sterile distilled water was added, the contents were vortexed, and the sand was allowed to settle. Microscopic observation at this stage demonstrated floating hyphae, which were collected by transferring 2ml of supernatant to a clean eppendorf tube and the hyphae were pelleted via centrifugation. DNA was extracted from the resulting pellet with the REDExtract-N-Amp Tissue PCR kit (Sigma-Aldrich, Saint Louis, MO USA), following the manufacturers instructions. DNA extractions were diluted 1 in 10 in sterile, DNA-free water and stored at 4°C. 

We amplified sets of samples in two ways. First, we amplified 10 μL of diluted DNA extract in duplicate from all four ingrowth bags and pooled the duplicate PCR products by circle (hereafter referred as pooled samples). This resulted in 40 samples, two per tree, corresponding to the inner and the outer circle. For a subset of 6 trees (3 old and 3 young) we also amplified and sequenced each individual ingrowth bag separately. These samples were amplified in triplicate and PCR products were combined, yielding 48 samples, eight per tree (four from the inner circle and four from the outer circle). The comparison between individual and pooled bags allowed testing the effects of pooling samples when assessing fungal diversity.

We amplified the internal transcribed spacer (ITS), the universal DNA barcode for fungi [35]. Forward primers comprised the 454 Fusion Primer A-adaptor, a specific MID barcode, and the ITS1F primer [36], while the reverse primer was composed of the B-adaptor and ITS4 primer [37]. Samples were amplified with unique 10 bp MID barcode to allow separating sequences according to sample in downstream analyses. 

Pyrosequencing PCR mixtures contained 1 unit of HotStarTaq polymerase (Qiagen, Valencia, CA), 2.5 μL of 1x PCR buffer supplied by manufacturer, 2.5 μL 10x each dNTPs (200uM), 0.5μL of 50 μM of both primers, 2 μL DNA template (all DNA extracts were diluted 1:10 to overcome inhibitors), and water to 25 μL. Following an initial denaturation at 95 °C for 15 minutes to activate the polymerase, samples were amplified by 35 cycles of 94 °C for 1 minute, 51 °C for 1 minute, 72 °C for 1 minute and subject to a final extension at 72 °C for 10 minutes. Replicate PCR products were combined and cleaned using AMPure magnetic beads (Beckman Coulter Genomics, Danvers, MA).

Amplicon samples were quantified using the Qubit flourometer (Invitrogen, Carlsbad, CA, USA) and pooled to an equimolar concentration. Sequences were run on 1/8th of a 454 FLX Titanium pico-titer plate at the Duke Institute for Genomic Sciences and Policy (Durham, NC, USA) and submitted to the National Center for Biotechnology Information (NCBI) Sequence Read Archive under accession number SRR768726. 

### Sequence analyses

Pyrosequences were processed using a QIIME pipeline (version 1.5.0) [38]. Sequences were assigned to individual samples, trees and circles, trimmed to lengths between 150 and 600bp and all reads including primer mismatches were removed to ensure accurate assignment of reads to samples. The resulting sequences were split into three equal batches (containing approximately 30,000 sequences each) and were denoised within QIIME. Then the ITS1 region was extracted from the denoised sequences using ITS Extractor [39]. The resulting reads were checked for chimeras and clustered into 95% similarity operational taxonomic units (OTU) using the USEARCH algorithm in QIIME. This lower threshold for OTU delineation was used because we focused only on the spacer region – structural gene sequences of the 18S and 5.8S genes were not considered. All singletons (reads found only once in the whole data set) were excluded from further analyses. In order to guarantee the best possible OTU identification, representative OTUs were searched against two in-house ITS reference databases using the BLAST+ algorithm [40]. The first database was a customized version of UNITE + INSDC (http://unite.ut.ee/repository.php) [41] containing additional ITS sequences from Californian identified voucher specimens that enabled greater accuracy in identification for fungi from our field site. The second database was a compilation of all fungal ITS sequences from the Point Reyes area deposited in GenBank. The BLAST+ output was further filtered to retain only BLAST+ alignments of >190bp in order to avoid fungal misidentifications. We retained the best BLAST outputs, i.e. the most complete identifications, and compiled an OTU table, including all identified OTUs and respective read abundances (Table S1). Representative sequences for the different OTUs can be found in Table S2 and a list of all OTU identifications on Table S3.

 We split the OTU table in two ways: 1) pooled versus individual samples; 2) EM and non-EM fungi. The latter was achieved with an in-house script (Figure S2). This script used a list of EM genera (Table S4) and potential EM genera (that are known to include both EM and non-EM taxa, Table S5) compiled and extended from [42] to create OTU tables containing only known EM fungi, all potentially EM fungi, and all other OTUs (mostly saprotrophic fungi). Given the low percentage of potential EM taxa compared to overall diversity, we focused our analyses on the EM and non-EM OTU tables. 

 Four of the 20 trees sampled in this study were also investigated in an earlier study [43], providing the opportunity for comparing the EM fungal diversities retrieved in both studies. We did so by comparing the number and identity of EM OTUs detected in these trees. 

### Root density and soil analyses

In the fall of 2012 we collected one 664 cm^3^ soil core from each of the spots where we buried the ingrowth bags in order to quantify root density and perform soil analyses for all the sampled trees. Soil from each core was sieved with a 2 mm sieve. All roots were saved, dried at 65 °C, pooled by circle, and weighed. Given the small amounts of roots found in the soil cores, we used total root biomass as a surrogate for root density. Soils were combined by circle per tree and total nitrogen and organic carbon, available phosphorus, and pH were analyzed using standard methods at the University of California Davis Analytical Laboratory. 

### Statistical analyses

**Figure 1:**
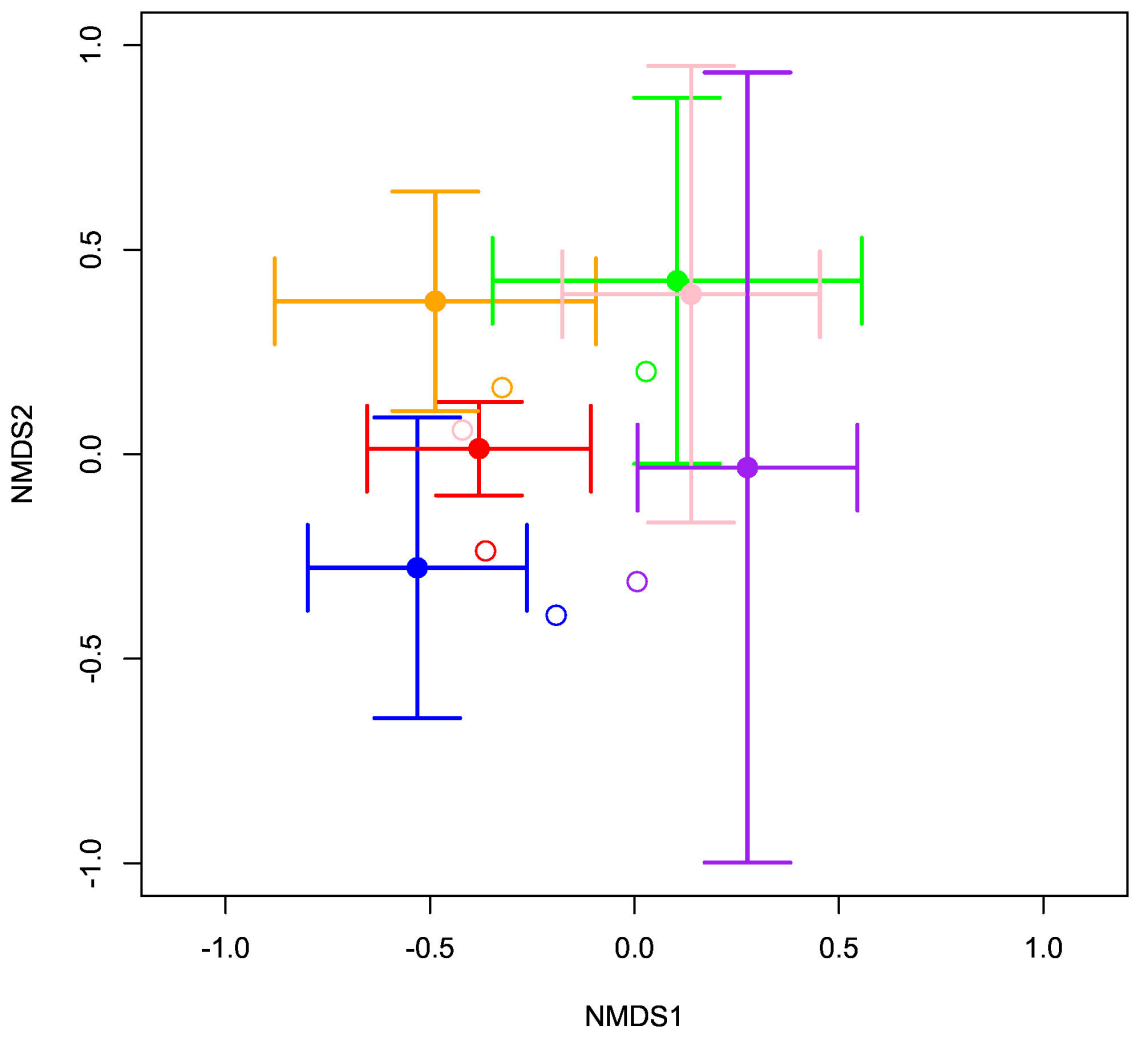
Comparison of individual versus physically pooled samples from six trees using NMDS ordination based on OTUs presence/absence (Stress = 0.25). Full circles and respective bars represent the centroids and two standard deviations of the fungal community recovered by individual bags from inner circles. The open symbols are the correspondent physically pooled sample. Each color indicates an individual tree. Individual and pooled ingrowth bags tend to recover different fungal communities, as pooled samples fall more often than not outside the two standard deviations of communities found in individual bags. This figure shows only the comparison of samples for inner circles. All other comparisons produced similar results and are not shown.

Our main goal was to determine whether there is zonation in fungal richness and composition under single tree canopies and across tree age. We plotted OTU accumulation curves to determine if sampling was adequate to fully describe the fungal assemblages associated with single trees; it was not (data not shown), so to allow valid comparisons we rarefied the data to 418 reads/sample in QIIME. We compared sampling methods for the 6 trees for which we had pooled and individual samples with a nested one-way analysis of variance to account for non-independence of samples collected under the same tree and by visualizing differences using non-metric multidimensional scaling (NMDS) based on the Bray-Curtis index of community dissimilarity (for both binary data and read abundance) [44]. We plotted the centroids and two standard deviation intervals for all individual samples from inner and outer circles and young and old trees, as well as the correspondent pooled sample. Sampling method was considered equally effective whenever a pooled sample fell within the correspondent two standard deviation of the individual samples (Figure 1). Both presence-absence and read abundance data produced similar trends, so we report only the former. In order to test for the effect of read depth, we also computationally pooled all OTUs recovered in the individual samples (per circle and tree) and compared these with the physically pooled samples. Samples were rarified to 98 reads/sample and potential composition differences were tested with ADONIS [44,45] and visualized using NMDS (as above). OTU richness between pooled and individual samples was compared with nested one-way analysis of variance, both before and after computational pooling of individual samples. These procedures allowed for further testing of effects of read depth on fungal community description.

We used two-way analysis of variance to test for significant differences in OTU richness across tree age and circle (inner/outer) for the pooled bags and nested two-way ANOVAs for individual bags (to account for non-independence of samples collected under the same tree). ANOVA assumptions of normality and homoscedascity were tested and Kruskal-Wallis tests were used whenever these assumptions were violated despite data transformation. OTU composition of each sample was also visualized with NMDS (as above) and tested for composition differences among all samples with ADONIS. Statistical analyses on both OTU presence/absence and OTUs weighted by read abundance data showed the same trends, so we report only the latter. Read abundances across circles and tree age were however used for assessing differences in selected taxonomic Orders using one-way ANOVA. Soil data was similarly compared across circle and tree age using ANOVA. The different soil parameters were fitted onto the ordinations to detect any potential gradient underlying fungal community structure. 

All statistical tests and graphics were performed using the program R version 2.15.1 (R Core Development Team, 2008). Distance matrices, NMDS ordinations and environmental fitting were performed with the Vegan package [44].

## Results

### Community overview

Out of the 96680 ITS1 sequence reads compiled in this study, 36% matched non-EM fungi, 18% EM fungi, 33% unidentified fungi, and 13% had no match in the searched databases. 4.3% of the reads were singletons and removed from the data set to lessen error and OTU inflation. We recovered a total of 368 fungal OTUs of which 70 (19%) were EM and 298 (81%) were non-EM (saprotrophic fungi for the most part). We found a diverse array of fungi belonging to Ascomycota (61%), Basidiomycota (31%), and Zygomycota s.l. (8%). The most frequently encountered taxa of each category (EM/non-EM) are given in Tables 1 and 2. The most frequent non-EM OTUs belonged to the genera *Fusarium, Mortierella*, and *Cladosporium*. Ten of the top 25 most common non-EM OTUs were found in over half the samples, and even the least common of the top 25 was found in almost a third of all samples (Table 1). The most frequent EM OTUs were *Tomentella sublilacina* and *Pseudotomentella atrofusca*. Thelephoraceae was the most highly represented Family with 23 OTUs (including OTUs in *Tomentella, Tomentellopsis*, and *Pseudotomentella*) and *Inocybe* was the most diverse EM genus with 11 OTUs. In contrast to the non-EM, the most common EM OTU were found in less than a third of all samples and 14 of the 25 (< 10%) most common OTUs were only found in four or less samples (Table 2). The controls revealed very little contamination, with three species present that were not found in the communities under study. 

**Table 1:** The 25 most frequent non-EM OTUs, with respective GenBank match, total percent occurrences, as well as percent occurrences in inner and outer circles, and young and old trees.

**OTUs**	**GenBank match**	**BLAST % match**	**Total (40)**	**Inner (20)**	**Outer (20)**	**Old (20)**	**Young (20)**
*Fusarium avenaceum*	KC464345	100	83	85	80	80	85
*Mortierella humilis*	JN943013	100	80	85	75	75	85
*Cladosporium cladosporioides*	AB693769	100	80	70	90	80	80
*Cryptococcus victoriae*	AJ581048	99	78	75	80	90	65
*Mortierella elongata*	JF439485	99	73	60	85	50	95
fungal endophyte	HE614871	99	63	70	55	60	65
*Leptosphaeria* sp.	HQ687895	100	58	35	80	55	60
uncultured fungus	GU559086	99	58	90	25	70	45
*Epicoccum nigrum*	AB693795	100	58	55	60	45	70
uncultured fungus	AB520280	98	53	50	55	65	40
*Boeremia exigua*	EU167567	98	48	50	45	35	60
uncultured fungus	JN905921	100	48	50	45	45	50
*Paecilomyces carneus*	JF311959	100	45	40	50	30	60
*Phialophora* sp.	GU004208	100	45	45	45	15	75
*Trichosporon porosum*	JN943732	100	45	40	50	65	25
*Cryptococcus* sp.	FR750602	100	43	45	40	30	55
uncultured fungus	JN032488	100	40	55	25	55	25
*Microdochium* sp.	JF424283	99	40	35	45	55	25
uncultured fungus	JN906629	99	40	30	50	30	50
Helotiales sp.	FR846484	100	38	25	50	35	40
*Mortierella* sp.	FJ861405	99	35	20	50	35	35
uncultured fungus	AB520569	100	35	25	45	25	45
Dothideomycetes sp.	EF619863	99	35	40	30	55	15
Agaricomycotina sp.	HQ212160	100	33	50	15	40	25
*Mortierella gamsii*	HQ630339	100	33	25	40	20	45

Numbers in parenthesis refer to the total number of samples for each category.

**Table 2:** 25 most frequent ectomycorrhizal (EM) OTUs, with respective GenBank closest match, total percent occurrences, and percent occurrences in inner circles, outer circles, young trees and old trees.

**OTUs**	**Genbank match**	**BLAST % match**	**Total (40)**	**Inner (20)**	**Outer (20)**	**Old (20)**	**Young (20)**
*Tomentella sublilacina*	DQ482015	100	28	35	20	30	25
*Pseudotomentella atrofusca*	EF619790	99	28	40	15	20	35
*Ceratobasidium* sp.*	DQ661903	100	23	15	30	35	10
*Amphinema* sp.1	GU180260	97	18	10	25	20	15
Thelephoraceae sp.1*	GU180329	100	18	25	10	10	25
*Russula sanguinea*	UC1859522	100	18	20	15	15	20
*Pseudotomentella* sp.	EF619810	99	15	20	10	15	15
*Tomentellopsis* sp.	FJ013094	97	15	25	5	20	10
*Geopora* sp.*	DQ822805	96	13	10	15	10	15
*Amphinema* sp.2	FJ552867	97	13	5	20	5	20
uncultured fungus*	GU180306	100	13	10	15	15	10
*Lactarius* cf. *luculentus*	DQ822819	100	10	20	0	15	5
uncultured Ascomycota*	FJ197199	99	10	15	5	5	15
*Tuber* sp.	HM021183	100	10	10	10	0	20
uncultured fungus*	AY702761	98	8	10	5	5	10
Thelephoraceae sp.*	EF411079	100	8	0	15	10	5
Helotiales sp.*	FJ827166	100	8	10	5	5	10
Thelephoraceae sp.2*	GU180327	100	8	5	10	10	5
*Thelephora* sp.	GU452529	100	8	5	10	5	10
*Inocybe* sp.	HM021165	100	8	10	5	10	5
*Suillus pungens*	HM021173	99	8	5	10	10	5
Trechisporales sp.*	JF519135	100	8	5	10	10	5
uncultured fungus*	HQ445530	99	8	0	15	10	5
*Russula amoenolens*	UC1859544	100	8	10	5	0	15
*Rhizopogon salebrosus*	UC1859714	99	8	10	5	15	0

Numbers in parenthesis refer to the total number of samples for each category. Unidentified OTUs marked with a * were good matches to fungi recovered from EM root tips in GenBank and therefore considered EM.

### EM fungi across circles and tree age

 The 40 physically pooled samples (including samples from inner and outer circles for each of the 20 sampled trees) revealed no significant differences in the number of EM OTUs across inner and outer circles (2-way ANOVA; *F* = 0.429, d.f. = 1, *P* > 0.05) or young and old trees (2-way ANOVA; *F* = 0.751, d.f. = 1, *P* > 0.05). EM composition was not different across circles (ADONIS, R^2^ = 0.03, *P* > 0.05) or young and old trees (ADONIS, R^2^ = 0.04, *P* > 0.05). The mean number of EM OTUs per outer circle per tree was 4, per inner circle per tree was 5, and per tree (combining circles) was 8.

**Figure 2:**
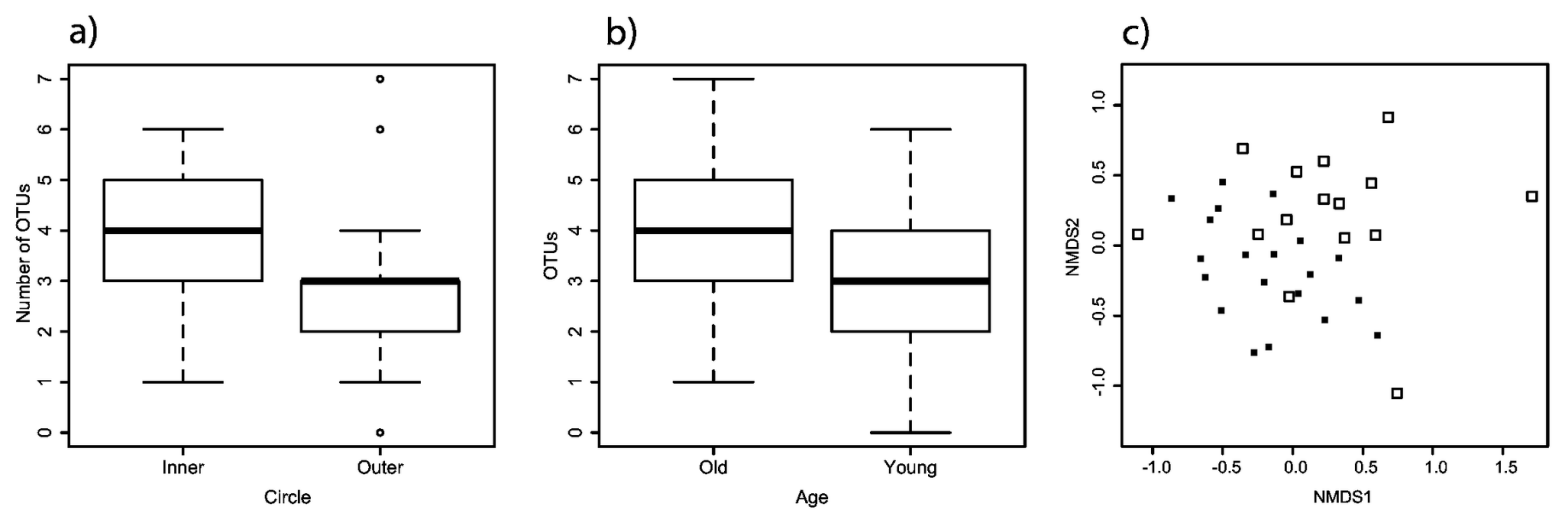
Boxplots showing ectomycorrhizal (EM) fungal diversity recovered from 48 individual ingrowth bags collected around six isolated *Pinus muricata* trees of two different ages. a) More EM OTUs in inner compared to outer circle (*P* < 0.05). b) More EM OTUs associated with old trees (*P* < 0.05). c) NMDS ordination based on OTUs presence/absence; each point represents an individual ingrowth bag, open squares are ingrowth bags from old trees, and closed squares are bags from young trees (stress = 0.1).

 The 48 individual samples from inner and outer circles from 3 old and 3 young trees revealed different patterns compared to pooled samples. There were significantly more EM OTUs in inner circles than outer circles (nested 2-way ANOVA: *F* = 6.31, d.f. = 1, 4, *P* < 0.05) and old trees compared to younger trees (nested 2-way ANOVA: *F* = 5.67, d.f. = 1,4 , *P* < 0.05) (Figure 2 a, b). EM fungal community composition was not significantly different between inner and outer circle (ADONIS, R^2^ = 0.04, *P* > 0.05), but was significantly different between young and old trees (ADONIS, R^2^ = 0.06, *P* < 0.05) (Figure 2 c), although the amount of variation explained was rather low. When computationally pooled, the mean number of OTUs per outer circle per tree was 8, per inner circle per tree was 14, and per tree (combining circles) was 22.

Comparisons of read abundance of EM fungal orders across circle and tree age (both pooled and individual samples) revealed only Boletales as significantly more prevalent in young trees compared to old (Kruskal Wallis, χ^2^ = 5.5, d.f. = 1, *P* < 0.05). This was driven by an increased presence of *Suillus* and *Rhizopogon* from soils surrounding young trees. 

We found 34 EM OTUs associated with the 4 pines trees that had been previously studied by [43]. Although this latter study was conducted in 2006 and was based on Sanger sequences obtained from single root tips, it provided very similar OTU richness (33 OTUs). However only five fungi were found in both studies (*Russula queleti, Russula sanguinea*, *Rhizopogon salebrosus*, *Tomentella sublilacina*, and *Tricholoma flavovirens*). *R. sanguinea* and *T. sublilacina* were common species in both studies, while the other three species were rare.

### Non-EM fungi across circles and tree age

**Figure 3:**
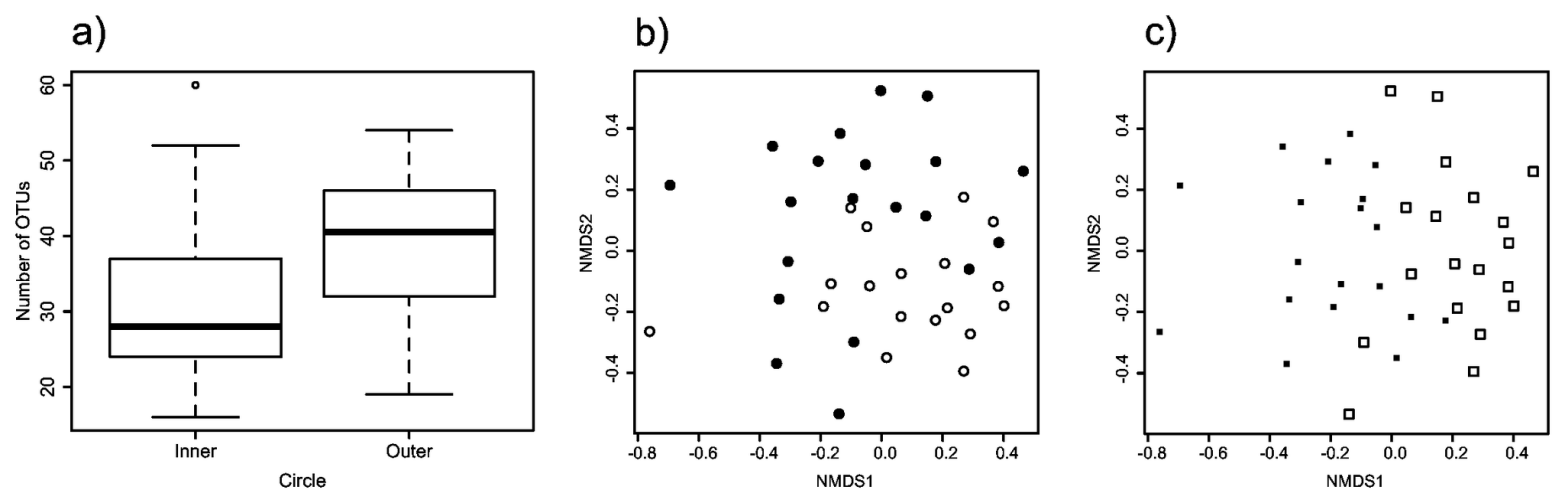
Boxplots showing non-ectomycorrhizal (non-EM) fungal diversity recovered from 160 pooled ingrowth bags collected around 20 isolated *Pinus muricata* trees of two different ages. a) More non-EM OTUs were found in outer than inner circles (*P* < 0.05). b) and c) NMDS ordination based on OTUs presence/absence; each point represents a set of four pooled ingrowth bags; open circles represent ingrowth bags from inner circles, closed circles outer circles, open squares old trees, and closed squares bags from young trees (stress = 0.27).

 Physically pooled samples showed more non-EM OTUs in outer circles compared to inner (2-way ANOVA; *F* = 5.29, d.f. = 1, *P* = 0.05) (Figure 3a), but there were no differences in richness across tree age (2-way ANOVA; *F* = 6.02, d.f. = 1, *P* > 0.05). The mean number of OTUs per outer circle per tree was 48, per inner circle per tree 38, and per tree (combining circles) was 81. Non-EM fungal composition was significantly different across circles (ADONIS, R^2^ = 0.07, *P* < 0.05) and tree age (ADONIS, R^2^ = 0.07, *P* < 0.05) (Figure 3 b, c), although the amount of variation explained was low. 

The basic pattern seen for non-EM fungi in individual bag samples was not different from the physically pooled bag samples except that the total number of OTUs was higher. Samples from individual bags contained more non-EM fungi in outer circles compared to outer circles (nested 2-way ANOVA; *F* = 5.46, d.f. = 1,4 , *P* < 0.05) (not shown), but no differences across tree age (nested 2-way ANOVA; *F* = 0.80, d.f. = 1,4 , *P* > 0.05). The mean number of OTUs per outer circle per tree was 86, per inner circle per tree 78, and per tree (combining circles) was 164. As in the pooled samples, saprotrophic fungal composition from individual bags was different across circles (ADONIS, R^2^ = 0.07, *P* < 0.05) and tree age (ADONIS, R^2^ = 0.07, *P* < 0.05).

### Pooled vs individual samples

We compared the total observed fungal species richness recovered from individual and physically pooled samples from the six trees. Individual samples yielded more OTUs than pooled samples (140 vs 90 OTUs on average; nested 1-way ANOVA, *F* = 49.61, d.f. = 1,2 , *P* < 0.05). NMDS showed that for four out of six trees, the composition of pooled samples for each tree fell outside the two standard deviations area defined by individual samples, indicating the two sampling strategies recover different views of assemblages (Figure 1). However, after computationally pooling individual samples and rarefying to the same sequence depth as the smallest pooled sample (98 reads), the community differences were no longer apparent. OTU richness was not significantly different, 23 OTUs for both individual and pooled bags on average (nested 1-way ANOVA, *F* = 0.07, d.f. = 1,2 , *P* > 0.05). Similarly, community structure was not significantly different between rarefied computationally pooled samples and physically pooled samples (ADONIS, R^2^ = 0.02, *P* > 0.05).

### Root density and soil chemistry

There were no significant differences in root density across inner and outer circle (Kruskal Wallis, χ^2^ = 0.12, d.f. = 1, *P* > 0.05) or old and young trees (Kruskal Wallis, χ^2^ = 1.2, d.f. = 1, *P* > 0.05). Soil analyses revealed significant differences in pH and C:N across circles and tree age. pH was higher in inner circles and old trees [mean pH(inner circle)=5.02, mean pH(outer circle)=5.4, mean pH(old trees)=5.0, mean pH(young trees)=5.4, 2-way ANOVA; *F* = 8.21, d.f. = 1, *P* < 0.05]. C:N was higher in inner circles than outer circles and in old trees compared to young trees [mean C:N(inner circle)=15.4, mean C:N(outer circle)=13.0, Kruskal Wallis, χ^2^ = 10.02, d.f. = 1, *P* < 0.05, mean C:N(old trees)=15.4, mean C:N(young trees)=13.0, Kruskal Wallis, χ^2^ = 11.9, d.f. = 1, *P* < 0.05]. However, fitting of soil chemical parameters onto the pooled sample ordinations mentioned above revealed that none of these parameters correlated with detected fungal community composition differences. 

## Discussion

Our results show the existence of small-scale partitioning in fungi associated with single trees. We report highly diverse and clearly distinct non-EM fungal communities in two canopy-defined zones under single trees and across tree age; these are spatial patterns that had not previously been reported at the community level. The two most obvious physical parameters that may drive this pattern are soil chemistry and moisture. 

 Soil moisture and chemistry have often been correlated with soil fungal community differences, with pH, C:N, and P as the most relevant parameters [19,46-49]. Soil properties around individual trees change in a gradient fashion from the trunk to the canopy line, with a general increase in pH and N [50,51]. Areas away from the trunk become less acidic due to differences in both stemflow and throughfall (the latter with lower pH compared to the former [51]). This was the case in our system, as we detected pH gradients that were consistent with these prior reports. Litter type (with bark litter more acidic than leaf litter; Frankland, 1998), and pH differences across canopy zones have been used to explain the preference for the outer canopy seen in *Mycena galopus*, a saprotrophic fungus growing under Sitka spruce trees [23]. Although this was a single species observation, it illustrates how saprotrophs can be influenced by or at least correlated with soil chemistry. N can also be relevant parameters for single tree soil zonation [23,50]. We found significant differences in C:N across circles and tree age, but not in N. We did not measure moisture disparities across the soil canopy-zones because this would have required repeated measurements over the season and access to these trees was difficult and time consuming. However in our fog-prone system moisture condensates around the tree canopies creating the so-called 'drip-line'. The ground under this outer edge of the canopy is noticeably wetter due to fog drip in the non-rainy period, which most likely affects fungal growth and establishment.

Interestingly, we did not find strong compositional zonation in EM fungi across soil canopy-defined zones or tree age, and those correlations that we did find were limited to circles with individual bags. As discussed below this may be partially due to the fact that EM fungi were a much smaller part of the detected OTUs per sample and the individual bags yielded greater sequence depth. EM fungal richness however was significantly higher in the inner circles with individual samples; this pattern is the opposite trend from that seen in non-EM fungi, and shows that EM and saprotrophic fungi respond differently to environmental conditions under single trees. Work by Talbot et al. [52] shows similar differences in response of EM and non-EM fungi to depth gradients and to organic detritus pools in closed forest systems in this same area. 

Peay et al. [31] proposed that EM fungi in forest islands are structured by a combination of host root density and fungal growth form (i.e. fungal exploration types [53]), and specifically hypothesized that lower root densities select for species with the ability to grow for long distances across the soil, while higher root densities select for species with shorter range growth strategies. Consequently, the outer edges of single trees and young trees that supposedly offer lower root densities would be colonized by long range exploration EM fungi, while inner canopies and old trees would have higher root density and would host a wide range of shorter range exploration EM fungi. We found little root biomass and failed to detect differences in root density across canopies and tree age. However, both long range (*Suillus*, *Rhizopogon*) and short exploration type species (*Inocybe*, *Lactarius*, *Russula*) were found in inner and outer circles as well as young and old trees, suggesting that low root density does not limit EM exploration types in this system.

Although pooled samples revealed no significant differences for EM fungi in either canopy-defined soil zone or tree age, this seems to be at least partially an artifact of sequence depth. We say this because the individual bags, which in combination provided approximately four-fold higher sequence coverage per zone per tree than pooled bags, did reveal EM fungal differences between zones and between different aged trees. However, when these data were computationally pooled and rarefied to the same sequence depth as the pooled bags the differences between the two datasets disappeared. In retrospect a higher sequence depth was probably needed to address the EM fungal patterns, but this was not obvious when the sampling scheme was designed, in part because we expected our sample to be dominated by EM fungal sequences. 

The very high abundance of non-EM fungi in our ingrowth bags contributed to the EM read depth problem. Ingrowth bags have been developed as a method to sample fungal mycelium in soil. They select for actively growing fungi and given their low organic content they were thought to be enriched in mycorrhizal fungi because these fungi are fueled by carbon sources from the tree host and do not require carbon from the substrate in order to continue growing. Previous studies reported that up to 30% of non-EM were detected using this method [6,34]. We, on the other hand, recovered a wide array of fungi, with EM comprising only 19% of the fungal communities in terms of number of OTUs and 30% in terms of sequence read abundance. Such discrepancy might derive from differences in bag size, since the bags used in this study were significantly smaller in comparison to previous studies (we used 5ml of sand compared to 30ml). Small sized bags have been recommended as they ensure a more uniform environment that is similar to surrounding soil and tend to be colonized faster [54]. However, small bags have a greater surface to volume ratio, and may better allow short, but frequent penetration from non-EM fungi. Furthermore, like any other sampling method, ingrowth bags have limitations and biases. The fungi captured in bags might not be a good representation of the mycelium available in the soil, as some groups of fungi may not colonize bags. *Cortinarius* spp. is a good example, since some of its species avoid mineral substrates [54], and in fact we found only two *Cortinarius* species in our samples despite their wide presence at the field site [33,43].

Despite the caveats, we captured a good representation of the Point Reyes National Seashore soil fungal diversity. Our EM fungal diversity is comparable to what has been described for the area, including some of the same species previously documented [11,31,33,43]. Our average of 5 EM taxa per bag is very similar to the number of OTUs found on roots in soil-core samples [43]. We also recovered a wide range of non-EM fungi (mostly saprotrophic), including many common soil fungi associated with the decomposition of plant material. Furthermore, finding small-scale structured spatial diversity in the saprotrophic community adds important information to this relatively poorly known group of fungi. Ingrowth bags provide a much less invasive method to sample fungi compared to soil cores and provide a view of active mycelial growth within a discrete time window. For these reasons we think they are a particularly useful tool for studying fungal communities, and the fact that our small bags do not discriminate strongly against non-EM fungi may expand, rather than limit, their utility. 

## Supporting Information

Figure S1 **Map showing the location of the sampled trees (Point Reyes National Shore).**
(TIFF)

Figure S2 **In-house script used to separate EM, potentially EM, and non-EM OTUs.**
(XLSX)

Table S1 **Complete OTU table for all fungi recovered in the study (with respective read abundance).**
(XLSX)

Table S2 **Representative sequences for all fungal OTUs.**
(XLSX)

Tables S3 **Fungal OTU numbering list.**
(XLSX)

Tables S4 **EM genera.**
(XLSX)

Table S5 **Potentially EM genera.**
(XLSX)
