# Supplementary figures and images for: Fungi at a Small Scale: Spatial Zonation of Fungal Assemblages around Single Trees
Source: PLoS One. 2013 Oct 16;8(10):e78295. doi: 10.1371/journal.pone.0078295 (PMC3797779; doi:10.1371/journal.pone.0078295)

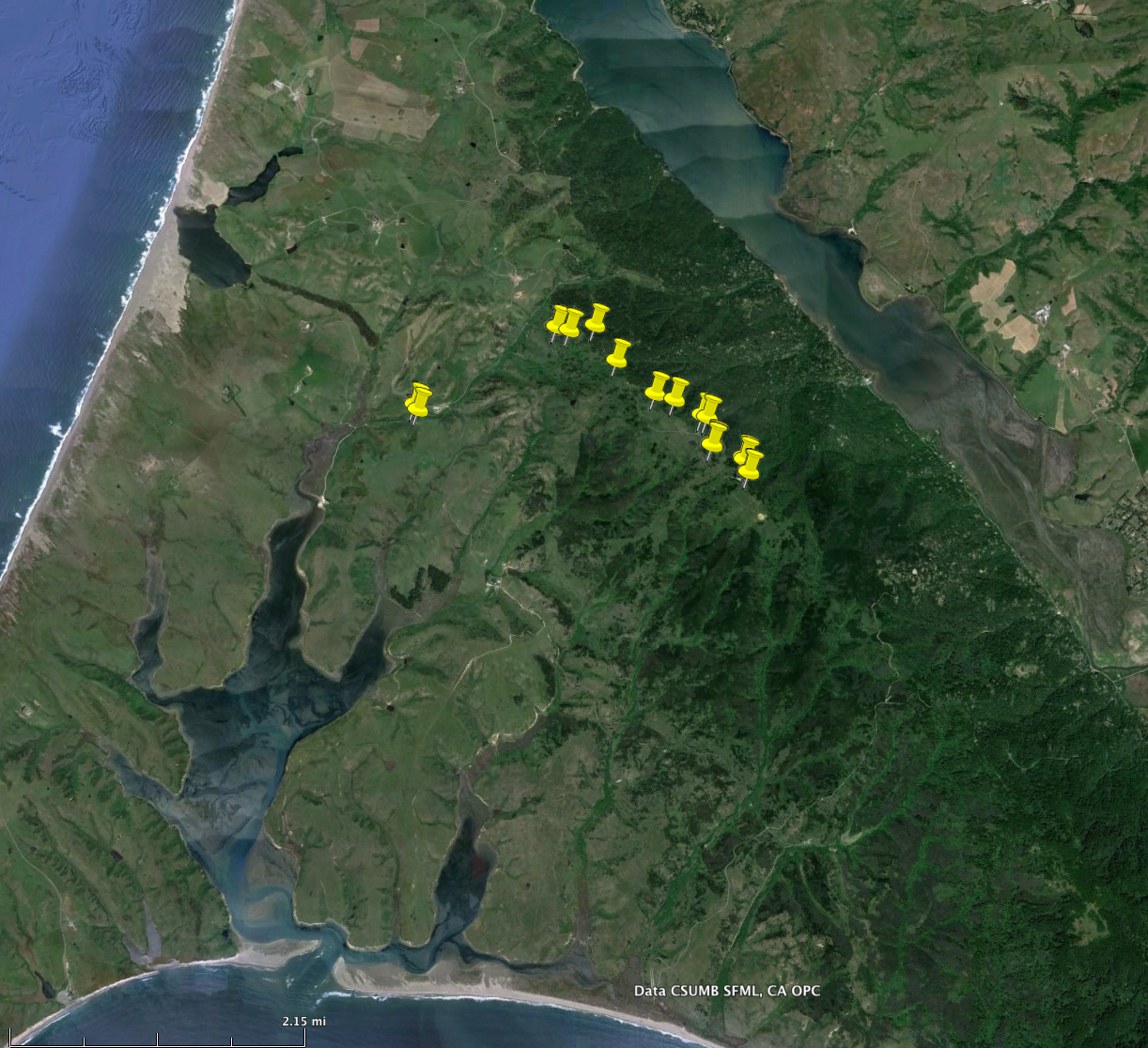

Supplement: Figure S1 — Map showing the location of the sampled trees (Point Reyes National Shore). (TIFF) [file pone.0078295.s001.tiff]
